# 

**Published:** 1816-11

**Authors:** 


### CATALOGUE OF MEDICAL BOOKS.

An Introduction to the Practice of Midwifery. By Thomas Denman, M.D. Fifth edition, improved. 8vo. 15s.

Treatise on Ruptures, containing an Anatomical Description of each Species, with an Account of its Symptoms, Progress, and Treatment. By William Lawrence, F.R.S. &c. &c. Third edition, with plates. 8vo. 14s.

Report of Observations made in the British Military Hospitals, in Belgium, after the Battle of Waterloo; with some Remarks upon Amputation. By John Thomson, M.D. F.R.S.E. &c. 8vo.

Experimental Outlines for a new Theory of Colours, Light, and Vision; with Critical Remarks on Sir Isaac Newton's opinions, and some new Experiments on Radiant Caloric. By Joseph Reade, M.D. &c. 8vo. Vol. I.

Some Practical Observations on Surgery, illustrated by Cases. By A. Copland Hutchison, Member of the Royal College of Surgeons, &c. 8vo. 7s.

Medical, Geographical, and Agricultural Report of a Committee appointed by the Madras Government to enquire into the Causes of the Epidemic Fever which prevailed in the Provinces of Coimbatore, Madura, Dindigal, and Tinnivelly, during the Years 1809, 1810, and 1811, of which Dr. W. Ainslie was President, Mr. A. Smith second Member, Dr. M. Christy third Member. 8vo. 6s. 6d.

Memoir on the Cutting Gorget of Hawkins, containing an Account of an Improvement upon that Instrument, and Remarks on the Lateral Operation for the Stone. By Antonio Scarpa. Translated from the Italian, by James Briggs, Surgeon, &c. 8vo.

Oracular Communications, addressed to Students of the Medical Profession; by Æsculapius. 12mo.

Sure Methods of attaining a Long and Healthful Life. By Lewis Cornaro. Translated from the Italian. 29th edit. 24mo.

An Essay on the common Cause and Prevention of Hepatitis, or Disorder of the Liver, and of Bilious Complaints in general, as well in India as in Europe: with an Appendix, recommending the old Submuriates of Mercury in preference to those now in use. By Charles Griffiths, M.D. Deputy Inspector of Hospitals, and late Senior Surgeon to the Forces. 8vo. 7s. boards.

Practical Illustrations of Typhus and other Febrile Diseases. By John Armstrong, M.D. 8vo. 10s. 6d.

Observations, with Cases, illustrative of a new, simple, and expeditious Mode of Curing Rheumatism and Sprains. By William Balfour, M.D. 8vo. 10s. 6d.

Extraordinary Case of Abscess in Perinæo, and other destructive Effects, in consequence of Stricture in the Urethra. By T. Foot. 8vo. 6d.

Accoucheur's Vade-Mecum. By J. Hopkins. Fourth edition. with additions and improvements. 12mo. 7s.

On the External, Chemical, and Physical Characters of Minerals. By Prof. Jameson. 8vo. 12s.

London Dissector, or System of Dissection practised in the Hospitals and Lecture Rooms of the Metropolis; explained by the clearest Rules, for the Use of Students: comprising a Description of the Muscles, Vessels, Nerves, and Viscera of the Human Body, as they appear on Dissection; with Directions for their Demonstration. Fifth edition. 12mo. 5s.

Synopsis Nosologiæ, or Outlines of a new System of Nosology. By T. M. D. Parkinson. Small 8vo. 7s.

A Treatise on Uterine Hæmorrhage. By Duncan Stewart, M.D. 8vo. 6s.

The Classes and Orders of the Linnæan System of Botany, illustrated by 240 plates of select Specimens of Foreign and Indigenous Plants. 3 vols. royal 8vo. coloured 7l. 4s.; plain 4l. 16s.

Improved Method of Treating Strictures in the Urethra. By T. Whately. New edition. 8vo. 7s.

---

### NOTICES TO CORRESPONDENTS.

*We earnestly entreat* Mr. A. *not to be offended if we venture to shorten his Letter. He will not suspect us of taking any other liberty; but, besides that, our readers are growing tired of the controversy—we are really fearful what may ultimately be the consequence, if longer protracted. The politeness hitherto preserved has scarcely led to a misunderstanding—to none, which may not be easily explained; and it must be admitted that many important truths have been elicited on both sides. In reality, we do not see that more need be said on the subject.*

*Communications have reached us from* R. Charnock, Esq., Mr. Beech, *and several others, which shall be duly attended to. We have also been favoured with a number of new publications.*

